# Aberrant DNA methylation in hepatocellular carcinoma tumor suppression (Review)

**DOI:** 10.3892/ol.2014.2301

**Published:** 2014-07-01

**Authors:** YOUHONG DONG, ANPING WANG

**Affiliations:** Oncology Department, Xiangyang Hospital Affiliated to Hubei University of Medicine, Xiangyang, Hubei 441000, P.R. China

**Keywords:** DNA methylation, tumor suppression, review, hepatocellular carcinoma

## Abstract

Aberrant DNA methylation leads to altered gene expression, resulting in cancerous features. Numerous tumor suppressor genes are silenced by DNA methylation during hepatocarcinogenesis. Promoter CpG island hypermethylation is an important mechanism for inactivating tumor suppressor genes in hepatocellular carcinoma (HCC). Hypermethylation of CpG islands in the p16 (INK4a) and p15 (INK4b) promoters may increase the risk of developing HCC, particularly hepatitis B virus-related HCC. Environmental factors can lead to geographic variations in the methylation status of CpG islands. Aberrant DNA methylation of CpG islands is catalyzed by DNA methyltransferases (DNMTs). Thus, abnormal variations of DNMTs can contribute to hepatocarcinogenesis. In hepatitis-related HCC, microRNAs participate in hepatocarcinogenesis by directly targeting DNMTs, during which hepatitis B virus X acts as a regulator. DNA methylation may also contribute to HCC tumorigenesis by regulating the cell cycle. Based on the importance of DNA methylation in tumor suppression of HCC, certain DNA methylations may predict the risk of tumor development, tumor staging, patient survival and HCC recurrence.

## 1. Introduction

Epigenetic mechanisms are important for human carcinogenesis. Epigenetic abnormalities are involved in the early stages of tumorigenesis and may trigger genetic events leading to tumor development (1). Epigenetic alterations result in aberrant gene expression profiles that do not result from changes in the primary nucleic acid sequence, but rather involve covalent modification of nucleotide bases in normal DNA sequences. DNA methylation is the most commonly studied epigenetic mechanism and is crucial in the development of nearly all types of cancer (2). Aberrant DNA methylation can result in altered patterns of gene expression leading to cancerous features. During carcinogenesis, numerous tumor suppressor genes are silenced by DNA methylation. DNA methylation does not change genetic information, however, alters the readability of the DNA and results in the inactivation of genes by subsequent repression of transcription. Tumors often possess decreased genomic DNA methylation levels and hypermethylated CpG islands (3).

Hepatocellular carcinoma (HCC) is the primary malignant tumor of the liver and the third leading cause of cancer-related mortality worldwide (4,5) Rising incidence and mortality rates from HCC have been observed in the majority of countries, particularly in Asia (6). During human HCC development and progression, DNA hypomethylation and regional CpG hypermethylation are dominant events (7). DNA methylation can occur as part of normal development, however, can also occur as a result of age or exposure to risk factors, potentially resulting in carcinogenesis in tissues with normal DNA sequences. HCC typically occurs in the setting of chronic inflammation that is secondary to the hepatitis B virus (HBV) or hepatitis C virus (HCV) infection, or alcoholism; each increases the risk for hepatocarcinogenesis. Furthermore, the HCV infection has been found to accelerate the methylation process in HCC (8).

Microarray analysis of HCC tissues has identified novel genes with cancer-specific methylation and 221 novel DNA methylation markers for HCC (9,10). TNFRSF10C, HOXA9, NPY and IRF5 were found to be frequently hypermethylated in HCC tissues and their methylation was identified to be closely associated with the inactivation of gene expression (9). In HCC cell lines, regional DNA methylation in tumor suppressor genes has been reported (11,12). The frequency of hypermethylation of tumor suppressor genes is relatively high in HCC, indicating that regional DNA hypermethylation is involved in hepatocarcinogenesis (13). In the present review, aberrant DNA methylation in tumor suppression of HCC is discussed.

## 2. Methylation hot spots on different chromosomes associated with early stage hepatocarcinogenesis

The epigenetic alteration of promoters by methylation is an alternative mechanism for the inactivation of tumor suppressor genes. Methylation hot spots on different chromosomes have been reported during early stage hepatocarcinogenesis. On chromosome 16, aberrant DNA methylation participates in the precancerous stage of hepatocarcinogenesis by preceding or causing loss of heterozygosity (14). At the D17S5 locus, aberrant DNA hypermethylation may participate in hepatocarcinogenesis during the early developmental stages and malignant progression of HCC (15). On chromosome 3, hypermethylation of multiple tumor suppressor genes, including Ras-association domain family 1, isoform A (RASSF1A), BLU and fragile histidine triad (FHIT), is a common and early event in hepatocarcinogenesis, as observed in human HCC tissues. Furthermore, CRBP1 methylation may be involved in later-stage carcinogenesis (16).

## 3. Methylation status of CpG islands

A CpG island is an ~1-kb DNA sequence with a high density of CpG dinucleotides and ~70% of human genes harbor CpG islands in their promoters (17,18). Promoter CpG island hypermethylation is an important mechanism for inactivation of tumor suppressor genes or tumor-related genes in human cancers and occurs in virtually all human cancer types (19). In a HCC rat model, the stages of multistage carcinogenesis following initiation are driven primarily by carcinogen-induced epigenetic alterations, including altered global histone lysine methylation patterns; increased histone H3 lysine 9 and histone H3 lysine 27 trimethylation in the promoter regions of the tumor suppressor genes RASSF1A, p16 (INK4a), suppressor of cytokine signaling (SOCS)1, E-cadherin 1 (CDH1)and Cx26, and early RASSF1A; and p16 (INK4a) promoter CpG island hypermethylation. These changes are accompanied by dysregulation of the balance between cell proliferation and apoptosis, a fundamental protumorigenic event in hepatocarcinogenesis (20).

Among the gene mutations mentioned above, p16 (INK4a) is important in regulating the cell cycle and mutations in p16 (INK4a) increase the risk of developing a variety of cancers. Adjacent to p16 (INK4a) is p15 (INK4b), which is also frequently mutated and deleted in numerous types of tumor; thus, p16 (INK4a) and p15 (INK4b) are candidates for putative tumor suppressor genes. In tumors of HCC patients from Japan, p16 (INK4a) was identified to be inactivated by extensive CpG methylation (21). However, tumors of HCC patients from Taiwan showed no aberrant 5′-CpG island hypermethylation of p16 (INK4a) or p15 (INK4b) in any primary tumors (22). The findings from different geographic regions vary. Environmental factors may affect the frequency and concordance of the degree of hypermethylation in multiple genes in HCC tumors, leading to the observed geographic variations in CpG island methylation status (23). CpG island methylation phenotype may be caused or facilitated by proliferative stimuli that are associated with environmental exposures. The precise mechanism of generating the CpG island phenotype requires investigation; however, the phenotype may contribute to screening, prevention or treatment of HCC in different geographic regions.

The HBV infection has a strong correlation with HCC occurrence (24–27) and aberrant CpG island methylation of genes has been recognized in hepatitis virus-related HCC. In studies regarding hepatitis virus-related HCC, Kiran *et al* (28) investigated promoter region methylation of a panel of six tumor suppressor genes: p16 (INK4a), p15 (INK4b), CDH1, glutathione S-transferase P (GSTP)1, SOCS1 and adenomatous polyposis coli (APC). The authors identified that the p15 (INK4b) methylation frequency and methylation allele density were higher in HCC than that in hepatitis (28). Furthermore, in HBV-associated HCC, the intensive hypermethylation of the CpG island of the tumor suppressor gene RASSF1A may be pathologically important in this tumor type, based on studies of human HBV-associated HCC tissues and HCC cell lines (Hep3B, HepG2, SK-HEP-1 and Huh-7) (29). In two HCC cell lines (HepG2 and Hep3B) RASSF1A can be inactivated and treatment of the cell lines with a DNA methylation inhibitor reactivates RASSF1A transcription (30).

A series of CpG island methylation alterations have been observed in the HCC cell lines Hep3B, HepG2, PLC/RPF/5/RPF/5, SMMC-7721, BEL-7402, MHCC97-H, MHCC97-L, HCCLM3 and HCCLM6. CpG island hypermethylation of tumor suppressor genes leads to a decrease in their expression (31,32).

## 4. DNA methyltransferases (DNMTs)

Aberrant DNA methylation on CpG islands is one of the most consistent epigenetic changes in human cancers and the methylation process is catalyzed by DNMTs. In mammals, five members of the DNMT family have been reported, DNMT1, DNMT2, DNMT3a, DNMT3b and DNMT3l. Among these proteins, only DNMT1, DNMT3a and DNMT3b exhibit methyltransferase activity. DNMT3a and DNMT3b establish methylation patterns at specific sequences, while DNMT1 maintains DNA methylation during replication by copying the methylation pattern of the parent DNA strand onto the newly synthesized strand (33,34). Abnormal variations of DNMTs participate in hepatocarcinogenesis.

In human hepatocarcinogenesis, DNMT1, DNMT3a and DNMT3b show a progressively increasing expression from normal liver, to chronic hepatitis/cirrhosis, to HCC (35). In the early and late stages of HCC development, global DNA hypomethylation and aberrant expression of DNMT1 and DNMT3b were identified in a glycine N-methyltransferase gene knockout mouse model for HCC (36). In a human HCC cell line, the depletion of DNMT3a suppressed cell proliferation and restored phosphatase and tensin homolog (PTEN), which is a crucial tumor suppressor in HCC. This indicated that PTEN may be the target of DNMT3a (37). Fan *et al* (38) observed a novel target of DNMT3b, metastasis suppressor 1 (MTSS1), which acts as a tumor suppressor in HCC. MTSS1 was repressed by DNMT3b via a DNA methylation-independent mechanism (38).

### Hepatitis-related HCC in the DNMT mechanism

The hepatitis B virus X (HBx) protein is involved in epigenetic modifications during hepatocarcinogenesis. Park *et al* (39) found that HBx repressed insulin-like growth factor-3 expression through *de novo* methylation via DNMT3a1 and DNMT3a2. Furthermore, HBx inhibited SP1 binding by recruiting methyl CpG binding protein 2 to a newly methylated SP1 binding element. HBx also induced global hypomethylation of satellite 2 repeat sequences by downregulating DNMT3b (39). In addition, the prevalence of these specific methylation abnormalities that are induced by HBx was identified to be significantly correlated with HBx expression in HBV-infected HCC patients (39). These findings indicated a potential association between DNMTs and HBV-infected HCC.

**Figure 1:**
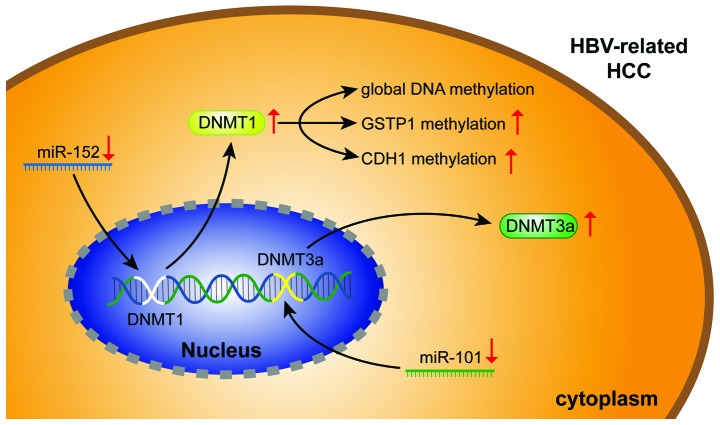
miR in HBV-related HCC. In HBV-related HCC, the inhibition of miR-152 resulted in global DNA hypermethylation and increased the methylation levels of two tumor suppressor genes, GSTP1 and CDH1. miR-101 was also downregulated and induced aberrant DNA methylation by targeting DNMT3a. miR, microRNA; HBV, hepatitis B virus; HCC, hepatocellular carcinoma; GSTP, glutathione S-transferase P; CDH1, E-cadherin 1; DNMT, DNA methyltransferase.

MicroRNAs (miRs) have also been identified to participate in the regulation of abnormal DNA methylation status in HBV-related HCC. By combining with the 3′-noncoding region of corresponding target mRNAs, miRs act as potent negative regulators of protein translation by disrupting mRNA stability, which affects the post-transcriptional regulation of genetic expression and is physiologically important (40). Zhang *et al* (41) identified that the expression of miR-152 was downregulated in the livers of HBx transgenic mice compared with the livers of wild-type mice. The authors also investigated the function of miR-152 as a tumor suppressor in epigenetic aberrations of HBV-related HCC (42). In HCC cell lines, the forced expression of miR-152 resulted in a marked reduction in the expression of DNMT1 by directly targeting the 3′-untranslated regions of DNMT1, which in turn led to a decrease in global DNA methylation. Inhibition of miR-152 resulted in global DNA hypermethylation and increased the methylation levels of two tumor suppressor genes, GSTP1 and CDH1 (42). miR-101 was also reported to be downregulated by HBx and to induce aberrant DNA methylation by targeting DNMT3a (43). Thus, miRs may participate in hepatocarcinogenesis by directly targeting DNMTs, during which HBx may act as a regulator (Fig. 1). miRs with key roles in regulation may be potential targets for inhibiting the development of HBV-related HCC.

### Reactive oxygen species (ROS) and the DNMT mechanism

In addition to being involved in inflammatory stimuli and associated proliferative changes associated with HBV, oxidative damage, which is associated with chronic inflammation directly affects the methylation status of DNA via DNMTs in HCC. ROS increase Snail expression, which recruits histone deacetylase 1 and DNMT1, and induces hypermethylation of the CDH1 promoter (44). Since CDH1 is a regulator of the epithelial-to-mesenchymal transition, this result is potentially relevant to understanding the activity of ROS in silencing tumor suppressor genes, and in subsequent tumor progression and metastasis. ROS accumulation mediates signal transduction cascades, and the activation of stress kinases and phosphorylation of substrates (45–47). Various studies have shown reduced histone deacetylases (HDAC) activity during oxidative stress (47–49) and in HCC, the expression of HDACs is associated with the HCC grade (50). In HCC cell lines, deacetylase inhibitors exert a dual effect on DNMT activity and expression, with rapid inhibition of enzyme activity from interference with post-translational acetylation and a delayed effect on transcriptional control of DNMT genes by HDACs or miR mechanisms (51).

## 5. DNA methylation in the cell cycle

DNA methylation contributes to HCC tumorigenesis by regulating cell proliferation. For example, DNA methylation of the promoter region of a candidate SRY box-containing gene 17 is found in 82% of HCC tissues and is associated with nuclear accumulation of β-catenin (52). β-catenin is an indispensable component of the canonical WNT signaling pathway (53) and is involved in cell differentiation, migration and proliferation during embryonic development and adult homeostasis (5). In HCC cell lines, the expression of family with sequence similarity 43, a novel tumor suppressor gene, reduced cell growth and colony formation *in vitro*, delayed the cell cycle and regulated DNA methylation (54). These findings indicated the involvement of DNA methylation in HCC by regulating cell proliferation.

**Figure 2:**
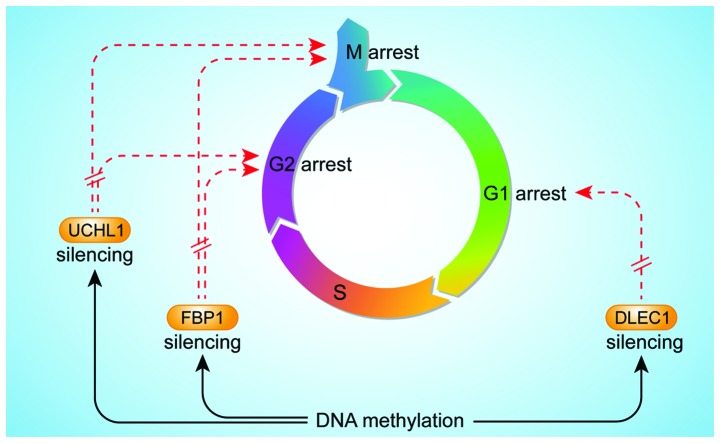
DNA methylation in the cell cycle. In hepatocellular carcinoma, the tumor suppressor gene, DLEC1 induces a G1 arrest in the cell cycle, while tumor suppressors, UCHL1 and FBP1 result in cell cycle arrest in the G2/M phase. When these genes are silenced by DNA methylation, their functions are inhibited. DLEC1, deleted in lung and esophageal cancer 1; UCHL1, ubiquitin carboxyl-terminal hydrolase L1; FBP1, fructose-1,6-bisphosphatase-1.

The cell cycle consists of four distinct phases, G1, S, G2 and M. In HCC cell lines, the tumor suppressor gene, deleted in lung and esophageal cancer 1 (DLEC1) decreases cell growth and cell size, and induces G1 arrest in the cell cycle, whereas DNA methylation silences DLEC1 (55). A comparable effect of DNA methylation on ubiquitin carboxyl-terminal hydrolase L1 (UCHL1) and fructose-1,6-bisphosphatase-1 (FBP1) indicates that they are tumor suppressors. UCHL1 silencing is reversed by genetic demethylation of the promoter, indicating direct epigenetic silencing. Restoring UCHL1 expression in silenced cell lines significantly inhibits their growth and colony formation ability, by inhibiting cell proliferation through cell cycle arrest in the G2/M phase and inducing apoptosis through the intrinsic caspase-dependent pathway (56). In addition, promoter hypermethylation mediates downregulation of FBP1 in human HCC, whereas restoring the FBP1 expression in the cells in which FBP1 expression is low significantly inhibits cell growth through the induction of G2-M cell cycle arrest (57) (Fig. 2).

In addition to the tumor suppressor genes mentioned above, DNA methylation is involved in silencing the expression and function of mac25/insulin-like growth factor binding protein-7, methylthioadenosine phosphorylase and TMEM7 in HCC (58–60).

## 6. Clinical applications

### Patient prognosis

The accumulating evidence for DNA methylation of tumor suppressor genes in HCC presents a potential clinical benefit. First, DNA methylation of tumor suppressor genes may aid in predicting the individual patient risk of tumor development. In chronic HCV patients, the methylation frequency of tumor suppressor genes, such as HIC1, GSTP1, SOCS1, RASSF1, CDKN2A, APC, RUNX3 and PRDM2 are associated with shorter time-to-HCC, and the number of methylated genes is an independent risk factor for HCC (61). These results indicate that characteristic patterns of altered DNA methylation are critical for the earliest steps of hepatocarcinogenesis and may predict the emergence of human HCC in HCV patients (61). Second, DNA methylation of tumor suppressor genes is associated with tumor biological features. For example, DLEC1 methylation is associated with the American Joint Committee on Cancer tumor staging (55).

Third, the DNA methylation status of tumor suppressor genes is valuable as a prognostic indicator in HCC patients. Calvisi *et al* (7) analyzed the global levels of DNA methylation and the methylation status of 105 putative tumor suppressor genes and identified that the extent of genome-wide hypomethylation and CpG hypermethylation correlated with the clinical outcome of HCC patients. The promoter DNA methylation of the Klotho gene was a predictive factor for poor HCC prognosis (62). Univariate and multivariate survival analysis revealed that HIST1H2AE methylation status is closely correlated with overall survival (10). The increased expression of DNMT3a and DNMT3b is suggested to be a predictor of poor HCC survival (35). MGMT methylation is considered to predict a shorter disease-free survival time (63), however, the sample sizes of these studies were small. Further studies with larger samples may aid in selecting the predictors of HCC survival.

The retinoblastoma protein-interacting zinc finger (RIZ1) gene performs tumor suppressor activity and is frequently silenced in numerous human cancers, including HCC (64–67). Promoter methylation of RIZ1 and H3K9 modifications act together in HCC to silence the RIZ1 gene, which is involved in HCC tumorigenesis, particularly in the early stage of the disease (68,69). Comparative analysis of promoter methylation and gene expression endpoints between tumor and non-tumor tissues from HCV-positive patients with HCC showed that RIZ1 methylation and increased levels of LINE-1 hypomethylation in non-tumor tissues are associated with time to recurrence. This underscores the importance of assessing the epigenetic state of liver remnants (70). In addition to RIZ1, methylation of CpG sites of the potential tumor suppressors, CFH and MYRIP, is associated with HCC recurrence (71).

### Clinical testing

Methods that are based upon DNA methylation patterns are useful in clinical testing for HCC. Iyer *et al* (72) compared tumor methylation profiles for the tumor suppressor genes APC, FHIT, p15, p16 and CDH1 in tumor tissues and plasma, and found that plasma DNA can be used for the reliable assessment of methylation profiles in HCC patients in an Egyptian population (72). To assess the medical applicability of CpG methylation as a molecular marker for cancer diagnosis, Kimura *et al* (73) established a novel system to determine DNA methylation based on TaqMan polymerase chain reaction combined with a methyl-binding-domain polypeptide 2. The availability of DNA methylation profiles for cancer diagnosis enable clinical predictions to be made from pre-therapy biopsies, paraffin-embedded samples or plasma DNA.

## 7. Conclusion

Aberrant DNA methylation results in altered gene expression, leading to cancerous features. During hepatocarcinogenesis, numerous tumor suppressor genes are silenced by DNA methylation. Hypermethylation of promoter CpG islands is an important mechanism for inactivating tumor suppressor genes in HCC. Although promoter CpG island hypermethylation of p16 (INK4a) and p15 (INK4b) may increase the risk of developing HCC, individuals from different geographic regions exhibit different methylation statuses for CpG islands. Aberrant DNA methylation of CpG islands are catalyzed by DNMTs, thus, abnormal variations of DNMTs may participate in hepatocarcinogenesis. Aberrant CpG island methylation of genes and DNMTs is involved in hepatitis-related HCC. miRs and ROS may participate in hepatocarcinogenesis by directly targeting DNMTs. Furthermore, DNA methylation may contribute to HCC tumorigenesis by regulating the cell cycle. Based on the importance of DNA methylation in tumor suppression in HCC, particularly the patterns of DNA methylation, it may predict the risk of tumor development, tumor staging, patient survival and HCC recurrence.

## Abbreviations

HCC: hepatocellular carcinoma
HBV: hepatitis B virus
DNMTs: DNA methyltransferases
HCV: hepatitis C virus
HBx: hepatitis B virus X
miR: microRNA
GSTP: glutathione S-transferase P
CDH1: E-cadherin 1
ROS: reactive oxygen species
UCHL1: ubiquitin carboxyl-terminal hydrolase L1
FBP1: fructose-1,6-bisphosphatase-1

## References

[1] Dumitrescu RG (2009). Epigenetic targets in cancer epidemiology. Methods Mol Biol.

[2] Jaenisch R, Bird A (2003). Epigenetic regulation of gene expression: how the genome integrates intrinsic and environmental signals. Nat Genet.

[3] Liu WR, Shi YH, Peng YF, Fan J (2012). Epigenetics of hepatocellular carcinoma, a new horizon. Chin Med J (Engl).

[4] Logan CY, Nusse R (2004). The Wnt signaling pathway in development and disease. Annu Rev Cell Dev Biol.

[5] Kim M, Lee HC, Tsedensodnom O (2008). Functional interaction between Wnt3 and Frizzled-7 leads to activation of the Wnt/betacatenin signaling pathway in hepatocellular carcinoma cells. J Hepatol.

[6] Parkin DM, Bray F, Ferlay J, Pisani P (2005). Global cancer statistics 2002. CA Cancer J Clin.

[7] Calvisi DF, Ladu S, Gorden A (2007). Mechanistic and prognostic significance of aberrant methylation in the molecular pathogenesis of human hepatocellular carcinoma. J Clin Invest.

[8] Nishida N, Nagasaka T, Nishimura T, Ikai I, Boland CR, Goel A (2008). Aberrant methylation of multiple tumor suppressor genes in aging liver, chronic hepatitis, and hepatocellular carcinoma. Hepatology.

[9] Shin SH, Kim BH, Jang JJ, Suh KS, Kang GH (2010). Identification of novel methylation markers in hepatocellular carcinoma using a methylation array. J Korean Med Sci.

[10] Jung N, Won JK, Kim BH (2012). Pharmacological unmasking microarray approach-based discovery of novel DNA methylation markers for hepatocellular carcinoma. J Korean Med Sci.

[11] Huang J, Zhang YL, Teng XM (2007). Down-regulation of SFRP1 as a putative tumor suppressor gene can contribute to human hepatocellular carcinoma. BMC Cancer.

[12] Zhang C, Li H, Zhou G (2007). Transcriptional silencing of the TMS1/ASC tumour suppressor gene by an epigenetic mechanism in hepatocellular carcinoma cells. J Pathol.

[13] Park HJ, Yu E, Shim YH (2006). DNA methyltransferase expression and DNA hypermethylation in human hepatocellular carcinoma. Cancer Lett.

[14] Kanai Y, Ushijima S, Tsuda H, Sakamoto M, Hirohashi S (2000). Aberrant DNA methylation precedes loss of heterozygosity on chromosome 16 in chronic hepatitis and liver cirrhosis. Cancer Lett.

[15] Kanai Y, Hui AM, Sun L (1999). DNA hypermethylation at the D17S5 locus and reduced HIC-1 mRNA expression are associated with hepatocarcinogenesis. Hepatology.

[16] Zhang X, Li HM, Liu Z (2012). Loss of heterozygosity and methylation of multiple tumor suppressor genes on chromosome 3 in hepatocellular carcinoma. J Gastroenterol.

[17] Saxonov S, Berg P, Brutlag DL (2006). A genome-wide analysis of CpG dinucleotides in the human genome distinguishes two distinct classes of promoters. Proc Natl Acad Sci USA.

[18] Weber M, Hellmann I, Stadler MB (2007). Distribution, silencing potential and evolutionary impact of promoter DNA methylation in the human genome. Nat Genet.

[19] Baylin SB, Chen WY (2005). Aberrant gene silencing in tumor progression, implications for control of cancer. Cold Spring Harb Symp Quant Biol.

[20] Pogribny IP, Muskhelishvili L, Tryndyak VP, Beland FA (2011). The role of epigenetic events in genotoxic hepatocarcinogenesis induced by 2-acetylaminofluorene. Mutat Res.

[21] Matsuda Y, Ichida T, Matsuzawa J, Sugimura K, Asakura H (1999). p16 (INK4) is inactivated by extensive CpG methylation in human hepatocellular carcinoma. Gastroenterology.

[22] Lin YW, Chen CH, Huang GT (1998). Infrequent mutations and no methylation of CDKN2A (P16/MTS1) and CDKN2B (p15/MTS2) in hepatocellular carcinoma in Taiwan. Eur J Cancer.

[23] Shen L, Ahuja N, Shen Y (2002). DNA methylation and environmental exposures in human hepatocellular carcinoma. J Natl Cancer Inst.

[24] Marotta F, Vangieri B, Cecere A, Gattoni A (2004). The pathogenesis of hepatocellular carcinoma is multifactorial event. Novel immunological treatment in prospect. Clin Ter.

[25] Cougot D, Neuveut C, Buendia MA (2005). HBV induced carcinogenesis. J Clin Virol.

[26] Anzola M (2004). Hepatocellular carcinoma, role of hepatitis B and hepatitis C viruses proteins in hepatocarcinogenesis. J Viral Hepat.

[27] Barazani Y, Hiatt JR, Tong MJ, Busuttil RW (2007). Chronic viral hepatitis and hepatocellular carcinoma. World J Surg.

[28] Kiran M, Chawla YK, Kaur J (2009). Methylation profiling of tumor suppressor genes and oncogenes in hepatitis virus-related hepatocellular carcinoma in northern India. Cancer Genet Cytogenet.

[29] Zhong S, Yeo W, Tang MW (2003). Intensive hypermethylation of the CpG island of Ras association domain family 1A in hepatitis B virus-associated hepatocellular carcinomas. Clin Cancer Res.

[30] Schagdarsurengin U, Wilkens L, Steinemann D, Flemming P (2003). Frequent epigenetic inactivation of the RASSF1A gene in hepatocellular carcinoma. Oncogene.

[31] Zheng D, Liu BB, Liu YK (2008). Screening for differential methylation status by CpG island microarray in the hepatocellular carcinoma cell lines. Zhonghua Zhong Liu Za Zhi.

[32] Liu BB, Zheng D, Liu YK (2010). Array-based profiling of the differential methylation status of CpG islands in hepatocellular carcinoma cell lines. Oncol Lett.

[33] Jurkowska RZ, Jeltsch A (2010). Silencing of gene expression by targeted DNA methylation: concepts and approaches. Methods Mol Biol.

[34] Kim JK, Samaranayake M, Pradhan S (2009). Epigenetic mechanisms in mammals. Cell Mol Life Sci.

[35] Oh BK, Kim H, Park HJ (2007). DNA methyltransferase expression and DNA methylation in human hepatocellular carcinoma and their clinicopathological correlation. Int J Mol Med.

[36] Liao YJ, Liu SP, Lee CM (2009). Characterization of a glycine N-methyltransferase gene knockout mouse model for hepatocellular carcinoma: Implications of the gender disparity in liver cancer susceptibility. Int J Cancer.

[37] Zhao Z, Wu Q, Cheng J (2010). Depletion of DNMT3A suppressed cell proliferation and restored PTEN in hepatocellular carcinoma cell. J Biomed Biotechnol.

[38] Fan H, Chen L, Zhang F (2012). MTSS1, a novel target of DNA methyltransferase 3B, functions as a tumor suppressor in hepatocellular carcinoma. Oncogene.

[39] Park IY, Sohn BH, Yu E (2007). Aberrant epigenetic modifications in hepatocarcinogenesis induced by hepatitis B virus X protein. Gastroenterology.

[40] Bartel DP (2004). MicroRNAs, genomics, biogenesis, mechanism, and function. Cell.

[41] Zhang X, Liu S, Hu T, Liu S, He Y, Sun S (2009). Up-regulated microRNA-143 transcribed by nuclear factor kappa B enhances hepatocarcinoma metastasis by repressing fibronectin expression. Hepatology.

[42] Huang J, Wang Y, Guo Y, Sun S (2010). Down-regulated microRNA-152 induces aberrant DNA methylation in hepatitis B virus-related hepatocellular carcinoma by targeting DNA methyltransferase 1. Hepatology.

[43] Wei X, Xiang T, Ren G (2013). miR-101 is down-regulated by the hepatitis B virus x protein and induces aberrant DNA methylation by targeting DNA methyltransferase 3A. Cell Signal.

[44] Lim SO, Gu JM, Kim MS (2008). Epigenetic changes induced by reactive oxygen species in hepatocellular carcinoma: methylation of the E-cadherin promoter. Gastroenterology.

[45] Blackburn RV, Spitz DR, Liu X (1999). Metabolic oxidative stress activates signal transduction and gene expression during glucose deprivation in human tumor cells. Free Radic Biol Med.

[46] Lee YJ, Galoforo SS, Berns CM (1998). Glucose deprivation-induced cytotoxicity and alterations in mitogen-activated protein kinase activation are mediated by oxidative stress in multidrug-resistant human breast carcinoma cells. J Biol Chem.

[47] Rahman I, Marwick J, Kirkham P (2004). Redox modulation of chromatin remodeling: impact on histone acetylation and deacetylation, NF-kappaB and pro-inflammatory gene expression. Biochem Pharmacol.

[48] Adenuga D, Yao H, March TH, Seagrave J, Rahman I (2009). Histone deacetylase 2 is phosphorylated, ubiquitinated, and degraded by cigarette smoke. Am J Respir Cell Mol Biol.

[49] Ito K, Lim S, Caramori G, Chung KF, Barnes PJ, Adcock IM (2001). Cigarette smoking reduces histone deacetylase 2 expression, enhances cytokine expression, and inhibits glucocorticoid actions in alveolar macrophages. FASEB J.

[50] Quint K, Agaimy A, Di Fazio P (2011). Clinical significance of histone deacetylases 1, 2, 3, and 7, HDAC2 is an independent predictor of survival in HCC. Virchows Arch.

[51] Zopf S, Ocker M, Neureiter D (2012). Inhibition of DNA methyltransferase activity and expression by treatment with the pan-deacetylase inhibitor panobinostat in hepatocellular carcinoma cell lines. BMC Cancer.

[52] Jia Y, Yang Y, Liu S, Herman JG, Lu F, Guo M (2010). SOX17 antagonizes WNT/β-catenin signaling pathway in hepatocellular carcinoma. Epigenetics.

[53] Grigoryan T, Wend P, Klaus A, Birchmeier W (2008). Deciphering the function of canonical Wnt signals in development and disease: conditional loss- and gain-of-function mutations of beta-catenin in mice. Genes Dev.

[54] Xu X, Liu RF, Wan BB (2011). Expression of a novel gene FAM43B repressing cell proliferation is regulated by DNA methylation in hepatocellular carcinoma cell lines. Mol Cell Biochem.

[55] Qiu GH, Salto-Tellez M, Ross JA (2008). The tumor suppressor gene DLEC1 is frequently silenced by DNA methylation in hepatocellular carcinoma and induces G1 arrest in cell cycle. J Hepatol.

[56] Yu J, Tao Q, Cheung KF (2008). Epigenetic identification of ubiquitin carboxyl-terminal hydrolase L1 as a functional tumor suppressor and biomarker for hepatocellular carcinoma and other digestive tumors. Hepatology.

[57] Chen M, Zhang J, Li N, Qian Z, Zhu M, Li Q, Zheng J, Wang X, Shi G (2011). Promoter hypermethylation mediated downregulation of FBP1 in human hepatocellular carcinoma and colon cancer. PLoS One.

[58] Komatsu S, Okazaki Y, Tateno M (2000). Methylation and downregulated expression of mac25/insulin-like growth factor binding protein-7 is associated with liver tumorigenesis in SV40T/t antigen transgenic mice, screened by restriction landmark genomic scanning for methylation (RLGS-M). Biochem Biophys Res Commun.

[59] Berasain C, Hevia H, Fernández-Irigoyen J, Larrea E, Caballería J, Mato JM, Prieto J, Corrales FJ, García-Trevijano ER, Avila MA (2004). Methylthioadenosine phosphorylase gene expression is impaired in human liver cirrhosis and hepatocarcinoma. Biochim Biophys Acta.

[60] Zhou X, Popescu NC, Klein G, Imreh S (2007). The interferon-alpha responsive gene TMEM7 suppresses cell proliferation and is downregulated in human hepatocellular carcinoma. Cancer Genet Cytogenet.

[61] Nishida N, Kudo M, Nagasaka T, Ikai I, Goel A (2012). Characteristic patterns of altered DNA methylation predict emergence of human hepatocellular carcinoma. Hepatology.

[62] Xie B, Zhou J, Yuan L, Ren F, Liu DC, Li Q, Shu G (2012). Epigenetic silencing of Klotho expression correlates with poor prognosis of human hepatocellular carcinoma. Hum Pathol.

[63] Lou C, Yang B, Gao YT, Wang YJ, Nie FH, Yuan Q, Zhang CL, Du Z (2008). Aberrant methylation of multiple genes and its clinical implication in hepatocellular carcinoma. Zhonghua Zhong Liu Za Zhi.

[64] Nishida N, Nagasaka T, Nishimura T, Ikai I, Boland CR, Goel A (2008). Aberrant methylation of multiple tumor suppressor genes in aging liver, chronic hepatitis, and hepatocellular carcinoma. Hepatology.

[65] Piao GH, Piao WH, He Y, Zhang HH, Wang GQ, Piao Z (2008). Hyper-methylation of RIZ1 tumor suppressor gene is involved in the early tumorigenesis of hepatocellular carcinoma. Histol Histopathol.

[66] Lal G, Padmanabha L, Smith BJ, Nicholson RM, Howe JR, O’Dorisio MS, Domann FE (2006). RIZ1 is epigenetically inactivated by promoter hypermethylation in thyroid carcinoma. Cancer.

[67] Chadwick RB, Jiang GL, Bennington GA, Yuan B, Johnson CK, Stevens MW, Niemann TH, Peltomaki P, Huang S, de la Chapelle A (2000). Candidate tumor suppressor RIZ is frequently involved in colorectal carcinogenesis. Proc Natl Acad Sci USA.

[68] Piao GH, Piao WH, He Y (2008). Hyper-methylation of RIZ1 tumor suppressor gene is involved in the early tumorigenesis of hepatocellular carcinoma. Histol Histopathol.

[69] Zhang C, Li H, Wang Y (2010). Epigenetic inactivation of the tumor suppressor gene RIZ1 in hepatocellular carcinoma involves both DNA methylation and histone modifications. J Hepatol.

[70] Formeister EJ, Tsuchiya M, Fujii H (2010). Comparative analysis of promoter methylation and gene expression endpoints between tumorous and non-tumorous tissues from HCV-positive patients with hepatocellular carcinoma. Mutat Res.

[71] Yang JD, Seol SY, Leem SH, Kim YH, Sun Z, Lee JS, Thorgeirsson SS, Chu IS, Roberts LR, Kang KJ (2011). Genes associated with recurrence of hepatocellular carcinoma: integrated analysis by gene expression and methylation profiling. J Korean Med Sci.

[72] Iyer P, Zekri AR, Hung CW, Schiefelbein E, Ismail K, Hablas A, Seifeldin IA, Soliman AS (2010). Concordance of DNA methylation pattern in plasma and tumor DNA of Egyptian hepatocellular carcinoma patients. Exp Mol Pathol.

[73] Kimura N, Moribe T, Iizuka N, Miura T, Tamatsukuri S, Ishitsuka H, Hamamoto Y, Oka M (2009). Rapid and quantitative detection of CpG-methylation status using TaqMan PCR combined with methyl-binding-domain polypeptide. Clin Biochem.

